# Spaceflight-associated neuro-ocular syndrome (SANS): expert consensus on diagnosis and management

**DOI:** 10.1038/s41433-026-04651-6

**Published:** 2026-06-30

**Authors:** Jessie Gew, Yousef F. Hyder, Olivia Grech, Tyson Brunstetter, William Tarver, Sara S. Mason, C. Robert Gibson, James D. Polk, Victor S. Schneider, Mary van Baalen, James L. Mitchell, Andreas Yiangou, Susan P. Mollan, Alexandra J. Sinclair

**Affiliations:** 1 Metabolism and Systems Science, School of Medical Sciences, College of Medicine and Health, University of Birmingham, Birmingham, UK; 2 Department of Neurology, University Hospitals Birmingham NHS Foundation Trust, Birmingham, UK; 3 National Institutes of Health and Care Research (NIHR) Birmingham Biomedical Research Centre, Birmingham, UK; 4 National Aeronautics and Space Administration (NASA), Johnson Space Center, Houston, TX USA; 5 Aegis Aerospace, Houston, TX USA; 6 KBR, Houston, TX USA; 7 South Shore Eye Center, League City, TX USA; 8 NASA Headquarters, Washington, D.C. USA; 9 Academic Department Of Military Rehabilitation, Stanford Hall, UK; 10 Department of Ophthalmology, Queens University, Kingston, ON Canada

**Keywords:** Optic nerve diseases, Neurological disorders

## Abstract

Spaceflight-associated neuro-ocular syndrome (SANS) is a disorder primarily characterised by changes in the eye and optic nerve, but changes in brain features are also noted after spaceflight. SANS is caused by exposure to prolonged microgravity and at least one feature of SANS has been reported in 81% of National Aeronautics and Space Administration (NASA) astronauts. Early clinical features of SANS are observed in almost all individuals undergoing spaceflight, with the condition exacerbated by the duration of spaceflight. Knowledge of the features of SANS and the aetiology has rapidly grown since its first description in 2011. As international space agencies plan exploration beyond low Earth orbit and with the expansion of the commercial space sector, a publicly available consensus guide on diagnosis and potential management would be instructive to flight surgeons who manage SANS. Harmonising case identification will not only enhance clinical management but is also vital to set a framework for research into SANS. Here we collate and interpret the up-to-date phenotyping and clinical insight in SANS, with expertise from the leading SANS experts at NASA. A consensus on diagnosis and management with countermeasures is detailed. Space exploration is a rapidly evolving field and consequently, insights into SANS will undoubtedly grow and allow us to further refine our understanding of this condition.

## Introduction

Spaceflight-associated neuro-ocular syndrome (SANS) is an emerging disease entity occurring during long-duration spaceflight (LDSF). As the mission duration for astronauts has increased over the past decades, SANS is becoming increasingly recognised, most prominently noted during 6 to 12-month missions onboard the International Space Station (ISS) [1]. With plans for the National Aeronautics and Space Administration (NASA) to return to the moon and beyond, mission durations will extend significantly and the prevalence and severity of SANS will likely increase without the use of additional countermeasures. The most significant SANS risks that could occur during extended-duration spaceflight without countermeasures include hyperopic shifts in refractive error, central visual distortions and an acute visual field loss (e.g. enlarged blind spot). There have also been suggestions that cognitive changes could occur in SANS (as they are noted in other conditions of altered brain fluid dynamics) [1–3]. However, evaluation of cognition in astronauts has been reassuring that there was no systemic decline in cognitive function, but only transient minor changes in processing speed, visual working memory, sustained attention and risk-taking propensity were observed [4]. Additionally, it is not yet known if a threshold exists beyond which permanent damage to the eye may occur due to SANS.

Early evidence of SANS is noted in almost all individuals exposed to microgravity. Therefore, there is a clinical need to publish a guide for diagnosis and management for any duration of space flight, to harmonise care and research. Here we bring together expertise from SANS specialists at NASA, together with knowledge from experts managing terrestrial conditions akin to SANS. We provide guidance on diagnostic criteria and management, with interpretation of the current knowledge and potential long-term sequelae of SANS.

## Methods

A literature search was conducted on Embase (1974 to 12 November 2025) and Ovid MEDLINE(R) (1946 to 12 November 2025) for articles in English. The search terms included ‘spaceflight-associated neuro-ocular syndrome,’ ‘visual impairment intracranial pressure,’ ‘ophthalmic syndrome,’ ‘optic disc edema,’ and ‘choroidal folds,’ combined with ‘astronaut’ or ‘spaceflight.’ Initially, 329 articles were found in the primary search. 73 articles were excluded after reviewing titles and abstracts. 255 articles were reviewed in full text, with additional articles identified from reference searches. Given the limited number of individuals who have flown onto the International Space Station, there was significant overlap between the participants included in these studies. In addition, the latest SANS prevalence data were obtained from the NASA Lifetime Surveillance of Astronaut Health (LSAH) program, along with the contemporary SANS clinical practice protocols from the NASA Space Medicine Operations Division.

### Consensus knowledge in SANS

#### Ocular features

In 2011, Mader et al. published the original case reports of SANS from seven astronauts following LDSF missions onboard the ISS [1]. Of those astronauts, five developed a hyperopic shift between +0.50 and +1.75 Dioptres. On further assessment, six astronauts had abnormalities on eye examination, including optic disc oedema, folds in the choroid layer of the retina, flattening of the posterior eye, cotton wool spots and retinal nerve fibre layer thickening on optical coherence tomography (OCT) imaging. A decade later, in 2021, Mader et al. reported a follow-up of three of the original SANS cohort to investigate long-term impacts [5]. Although optic disc oedema had resolved in all three astronauts by six months after returning to Earth, the hyperopic shift, globe flattening and change in the eyes axial length had persisted for up to 12 years, indicating that some permanent structural changes had occurred following LDSF [5].

Enlarged blind spots within the visual field have been observed immediately post-flight in two crew members and one other crew member self-reported an in-flight visual scotoma that resolved before return to Earth [2]. In a self-reported questionnaire of three hundred astronauts, 60% of LDSF crew reported subjective changes in their visual acuity [1]. A study of fourteen astronauts and cosmonauts identified a mean decrease of 0.75 eye-chart lines in visual acuity during walking one day after returning from space [6]. In a separate study involving thirty-one astronauts, it was discovered that there was a transient 7–10% increase in the average time needed to visually acquire targets after landing [7].

Hyperopic shifts are common during spaceflight and ‘space anticipation glasses’ are routinely deployed with ISS crew members [8]. A prospective cohort study found 27/56 (48.2%) eyes experienced a hyperopic shift after LDSF. An average of +0.12D increase (95% CI + 0.02 to +0.22D) was observed [9]. Increased diameter of the optic nerve sheath has also been identified in some individuals [10], with findings documented as early as day 10 in space [11], but this has not been a consistent finding. Variability in optic nerve sheath measurement acquisition and known tolerances of the measure could be a reason for the uncertainty of this outcome. To utilise optic nerve sheath diameter measures as a meaningful sign in SANS, the precise methodology needs to be standardised and validated in clinical medicine.

Cotton wool spots, isolated retinal nerve fibre layer infarcts, have been observed in astronauts both during and following spaceflight [2]. Some have been observed for many years following spaceflight. Although not considered part of the SANS phenotype, their presence is still documented due to unclear aetiology.

Another subjective feature recorded in optic disc oedema that is challenging to study is the presence or absence of spontaneous venous pulsations at the optic nerve head [12]. Observation of spontaneous venous pulsations in terrestrial medicine is observer-dependent and thus contributes to the inconsistency of reporting in the context of LDSF.

The fidelity of OCT imaging has benefited the characterisation of SANS. A detailed examination of the OCT images has shown an increase in peripapillary total retinal thickness (TRT), which begins proximally around the optic nerve head and spreads radially [13] and occurs with a bias towards the nasal peripapillary retina [14]. An increase in global peripapillary choroid thickness [13, 15, 16], optic nerve head minimum rim width [13, 17] and retinal nerve fibre layer thickness [14] are reported in both males and females.

Comparison of crew members who served on short (≤30 days), long (>30 days) and extended (around 1 year) duration missions has shown that optic disc oedema and choroidal folds progressively increase with exposure to LDSF [18] and repeated missions lead to cumulative changes [10, 19]. Furthermore, globe flattening and anterior displacement of the optic nerve only occurred after LDSF [20]. It is not currently known if these abnormalities continue to progress or plateau. Table 1 shows the prevalence of ocular findings seen in astronauts following LDSF. 52 of 64 (81%) of NASA astronauts had at least one ocular finding of SANS. However, caution in interpretation is recommended with these historical data because of the different methodologies and technicians used to assess clinical features occurring during and post space flight. In addition, incidence of clinical features may be influenced by the difference between a ‘rookie’ astronaut (i.e., those on their first mission) and a ‘veteran’ astronaut on a repeat mission and to a large degree, most of the literature has yet to divide the prevalence of features based on this nuance.

**Table 1:** Ocular features of spaceflight-associated neuro-ocular syndrome (SANS) in NASA astronauts (July 2000 to May 2025) amongst 64 astronauts.

Features	Prevalence (number evaluated)
Optic disc oedema^a^	69% (48)
Chorioretinal Folds	25% (64)
Posterior globe flattening^b^	37% (43)
Refractive error^c^	20% (45)
Any SANS criteria^d^	81% (64)

Missing data occurs if an evaluation cannot be completed.

^a^This requires at least one postflight OCT scan to determine subclinical oedema

^b^This requires pre and postflight MRI

^c^This requires pre and postflight cycloplegic refractive error

^d^Based on crew member who met one of these criteria (a, b, c)

In summary, ocular anatomical changes have been recorded in the majority of astronauts during LDSF. No permanent functional changes have yet been recorded and no countermeasures beyond utilising higher-powered spectacles (i.e., space anticipation glasses) have been established. Future work should establish thresholds for commencing countermeasures and medical interventions before long-term changes occur and evaluation of terrestrial analogues to determine biomarkers of long-term negative outcomes.

#### Brain features: evolving manifestation of SANS

**Fig. 1:**
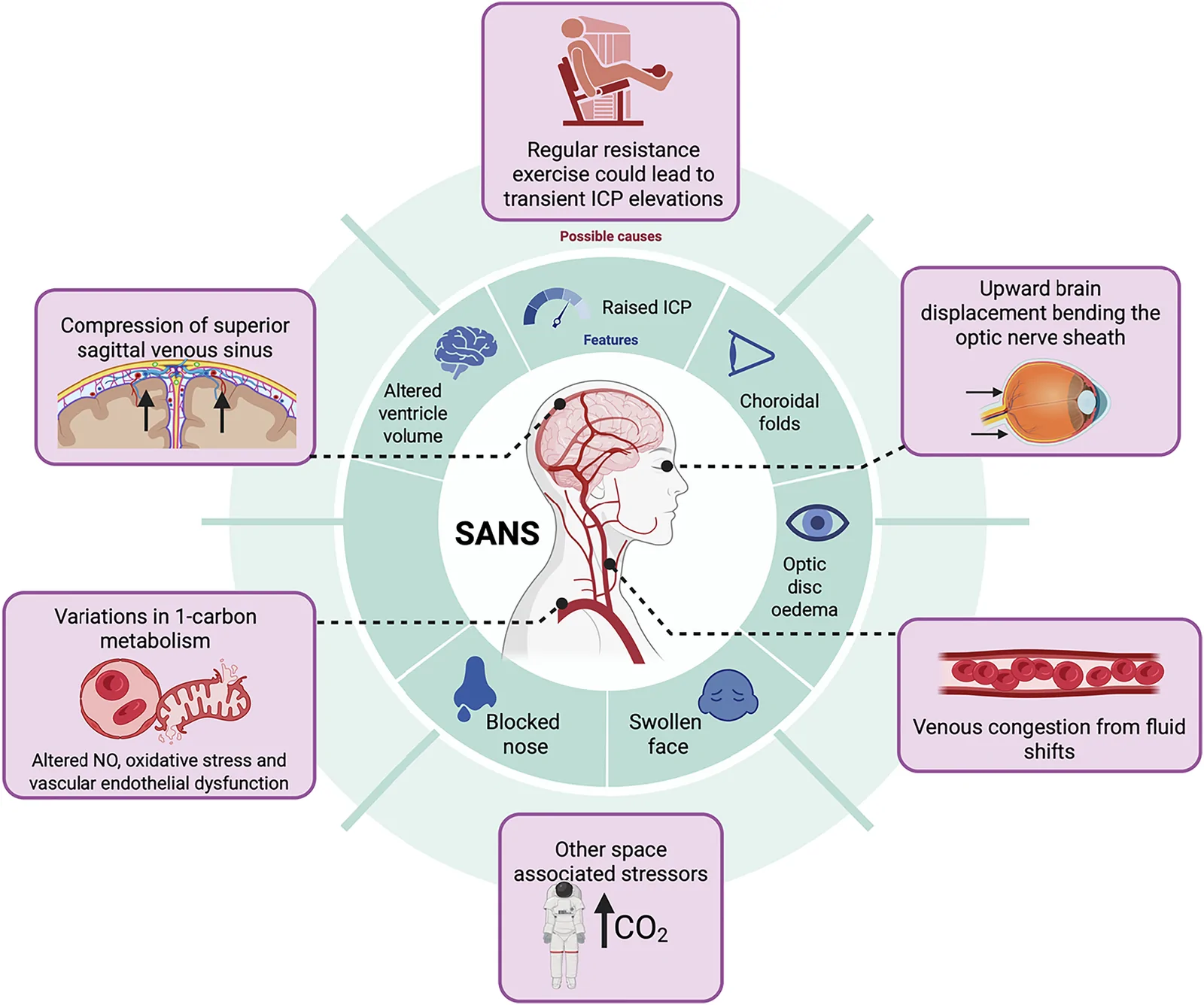
Infographic summarising features of spaceflight-associated neuro-ocular syndrome (SANS). The figure illustrates the key features of SANS and its contributing factors.

To date, unexplained changes have been documented in post-flight MRI scans in the brains of astronauts following LDSF, raising a possible concern for more widespread risks associated with spaceflight (Fig. 1). The precise relationship of the neuroimaging findings to the ocular features of SANS has not been established. Some features of SANS meet the diagnostic criteria for secondary pseudotumor cerebri syndrome (PTCS), including bilateral disc oedema, neuroimaging confirming no lesion causing raised ICP and where there is a direct causal factor causing the syndrome, LDSF. In astronauts, neuroimaging studies have shown minor expansions to the volume of the lateral and third ventricles [21], increased cerebrospinal fluid (CSF) stroke volume in the cerebral aqueduct and reduced cerebral compliance after LDSF [22, 23]. Although ventricular size remains within normal limits for age, the rate of increase is greater than expected [23–25].

Periventricular white matter hyperintense lesions have also been identified post LDSF, which could represent increased interstitial fluid secondary to increased ventricular volume. Structural changes of the left caudate nuclei, found adjacent to the lateral ventricles, were associated with impaired postural control after LDSF [26]. Other white matter microstructural changes as measured by diffusion anisotropy were identified in the cerebellum, superior and inferior longitudinal fasciculus and corticospinal tract in association with LDSF [27]. Reports of structural changes are highly variable and it is not clear if these represent a reliable and reproducible feature after LDSF [28], these results should be interpreted with caution.

Spaceflight-induced intracranial extracellular free water shifts have been documented between pre and post flight MRI [27]. An increase in MRI-visible perivascular spaces (PVS) in white matter has been demonstrated in novice astronauts [29]. No significant differences in PVS characteristics were found between those who did or did not exhibit symptoms of SANS. This finding is contradicted by another study that found white matter PVS volumes from before to after LDSF in astronauts who developed signs of SANS increased compared to those who did not [30]. However, there remains a lack of validated tools to quantify the changes in PVS.

Further support for neurological involvement in SANS is the presence of blood biomarkers indicative of neuronal injury. A study of five cosmonauts following LDSF found that blood biomarkers of neural injury, including neurofilament light chain (NfL), glial fibrillary acidic protein (GFAP), total tau and beta amyloid (Aβ40), were elevated [31]. Although cognitive dysfunction has not been reported specifically in SANS, cognitive impairment has been observed in terrestrial CSF disorders [3] and astronauts immediately following LDSF [32]. A study found subtle but significant changes in cognitive or sensorimotor performance that adversely affect operator proficiency among eight astronauts on the day of return from ISS missions and the cognitive impact was likely due to factors associated with spaceflight, rather than fatigue alone [33].

The correlation between the ocular and neurological findings remains uncertain. A prospective study observed an association of lateral ventricular volume changes with visual acuity difference in the left eye pre and postflight, but not in the right eye [24, 34]. In another study, a weak association between total retinal thickness and lateral ventricle volume was demonstrated [35].

In summary, both ocular and brain features have been observed in SANS. However, the small number of cases limits the reliability of any inferred correlation.

#### Potential role of intracranial pressure in SANS

The clinical manifestations of SANS can resemble those seen in disorders of raised intracranial pressure (ICP). An estimated increase in ICP of 6 mmHg after LDSF was interpreted from MRI scans of astronauts [36], due to characteristic morphological changes to the pituitary, including increased concavity, posterior displacement and reduced height of the pituitary stalk [23, 37]. However, ICP has never been directly measured in astronauts whilst in space. Doing so would expose otherwise healthy crew members to the risks of invasive ICP testing (such as lumbar puncture or telemetric ICP monitoring). Indeed, lumbar puncture would be technically challenging as spinal anatomy distorts in space and the standard water-column manometer relies on gravity to measure pressure [38]. Invasive ICP recordings in spaceflight from a macaque monkey showed an increase of 5 mmHg and reduced cerebral compliance on entering space, compared to the seated position on Earth [39].

Lumbar punctures performed on astronauts with optic disc oedema (i.e., SANS) after returning to Earth have measured opening pressures ranging between 18 and 28.5 cmH_2_O [1, 5, 10, 40, 41]. Of note, for terrestrial cases of optic disc oedema, a lumbar puncture opening pressure greater than 25 cmH_2_O is required to diagnose idiopathic intracranial hypertension (IIH) [42]. Notably, the timepoint of lumbar puncture measurements varied between astronauts, with the earliest measurements conducted in some individuals within 7 days of return, while others measured up to seventeen months afterward. During this time, re-exposure to Earth’s gravity likely ameliorated the pathophysiological processes underpinning SANS and any potential ICP disturbance.

More recently, non-invasive surrogate measures of ICP have been evaluated, such as cerebral and cochlear fluid pressure, otoacoustic emissions, ultrasound measures of the optic nerve sheath diameter (ONSD) and ultrasound-based internal jugular vein pressure [40]. As these non-invasive surrogate measures of ICP have not been validated against the gold standard invasive direct measures of ICP, the diagnostic and monitoring capabilities of these non-invasive tests remain uncertain and the established normative values are lacking.

To advance understanding of ICP dynamics under microgravity, studies have utilised parabolic flights, which offer brief periods of weightlessness lasting approximately 17–25 s, immediately followed by approximately +1.8 g as the aircraft regains altitude for the next 0 g cycle. Consequently, only short-term effects of microgravity can be assessed and the alternating exposure to hypergravity introduced a potential confounding variable. Notably, during the microgravity phase, while subjects were in a supine position, direct ICP measures via an intraventricular catheter system were reported to reduce by 4 mmHg compared to baseline measurement under normal gravity [43]. The relevance of the findings from parabolic flight to SANS and LDSF remains uncertain. Moreover, the utility of parabolic flight as a research modality for understanding ICP alterations in the context of LDSF is likely limited.

It is worth noting that ICP is lower when standing upright compared to when lying down [43–45]. These postural changes in ICP are lost in microgravity and are likely contributory to the brain fluid shifts observed in SANS. The terrestrial ICP alterations with posture are profound and illustrated in a prospective study of fifteen patients with idiopathic intracranial hypertension, which found that mean ICP decreased by 51% when moving from the supine position to standing [46]. In microgravity, it is assumed that the ICP remains constant regardless of whether the person is lying or standing. Therefore, it is hypothesised that SANS may originate from a slight increase in the average ICP without the typical fluctuations in moving between the upright and supine positions that are experienced on Earth. A subtle, persistent ICP rise may be sufficient to induce optic nerve oedema in the situation of microgravity [45, 47]. This may explain why astronauts do not report symptoms typically associated with markedly elevated ICP, such as chronic headaches, diplopia, transient visual obscuration or pulsatile tinnitus [48, 49], yet are still noted to have optic nerve oedema.

### Mechanisms of SANS

#### Changes in venous return

On entering microgravity, approximately two litres of fluid are redistributed upwards from the lower body veins, leading to symptoms of a congested nose and swollen face. Consequently, the internal jugular veins dilate and pressure within them increases [43, 50, 51]. Of concern, a very small number of crew members have been diagnosed with internal jugular vein thromboses, likely resulting from stagnant and/or reverse internal jugular vein blood flow [52–54].

The relationship between internal jugular vein thromboses and SANS ocular features remains undefined, particularly if this represents a complication of SANS or is a distinct diagnostic entity occurring during spaceflight. Post-flight MRI demonstrated enlarged intracranial venous sinuses in astronauts with signs of SANS [55], indicating potential impaired return of fluid from the head and neck. This may result in venous stasis around the eye and optic nerve, leading to retinal vascular remodelling, optic disc oedema and reduced CSF absorption [56, 57].

It has been proposed that venous stasis in microgravity may directly lead to choroidal engorgement and thickening [1, 2]. Like terrestrial pathology, such as central serous chorioretinopathy or peripapillary pachychoroid syndrome [58, 59], the choroid may experience venous congestion and excessive hydrostatic pressure during spaceflight, leading to a venous overload choroidopathy [59]. Spaceflight-induced choroidal changes may induce the hallmark signs of SANS, especially chorioretinal folds, as well as other ophthalmic signs diagnosed during LDSF (e.g. retinal pigment epithelial detachments).

#### CSF and glymphatic circulation

Microgravity may also inhibit normal CSF and glymphatic circulation and drainage. An upwards displacement of the brain has been documented on post-LDSF MRI, with reduced CSF at the vertex and increased CSF at the base of the cerebellum and lateral fissure [21, 26, 27, 34] and upward stretching of the pituitary stalk and optic chiasm [21]. Some authors have suggested that this upwards movement may disrupt nearby venous structures such as the superior sagittal sinus, which may result in limited CSF flow across its arachnoid granulations [21]. Ultrasound measurements conducted on the ISS show increased middle cerebral vein velocity, which is consistent with reduced cross-sectional area in microgravity [60]. Cerebral aqueduct rotation has been detected in certain astronauts, suggesting potential increased resistance to CSF outflow from the third ventricle [16]. However, further research is necessary to confirm this finding.

Potentially, the upward brain displacement may pull on the optic nerve sheath and generate a posterior force on the eye, which may contribute to globe flattening seen in astronauts [61, 62]. However, a recent prospective MRI report has shown that while upward displacement of the optic chiasm was common among LDSF astronauts, there was no correlation between this and the clinical manifestations of SANS. [63]. It should be recognised that a degree of recovery may have already occurred after landing prior to the MRI scan being performed, which may mask any association between the upward brain displacement and SANS.

Given that the degree of globe flattening and optic nerve head protrusion due to SANS has been correlated with an increase in optic nerve sheath volume [36], some authors have speculated that in microgravity, CSF sequesters within the narrow and septate optic nerve subarachnoid space in a mechanism akin to a one-way valve, leading to a compartment syndrome [64]. Hence, pressure in the optic nerve sheath may rise despite normal ICP. Consequently, CSF may be forced along perivascular spaces into the optic nerve and glymphatic outflow from the eye may be impeded, resulting in optic disc oedema [64, 65]. Variations in optic canal anatomy or optic nerve sheath compliance may result in greater pressure elevations in different individuals or between the eyes of a single individual [66, 67].

#### Carbon dioxide level

Several other environmental factors on the ISS have been proposed to contribute to SANS (Fig. 1). Ambient CO_2_ levels often rise above normal workplace limits on the ISS [68]. However, the levels of hypercapnia on the ISS have not increased arterial or end-tidal PCO_2_. Hypercapnia is known to cause cerebral vasodilation, leading to increased cerebral blood flow and potentially raised ICP [69]. CO_2_ levels on the ISS were found to be significantly associated with headache. The odds of a crew member reporting a headache doubled with each 1 mmHg increase in CO_2_. However, there was no association observed between the level of CO_2_ and visual changes or SANS [70]. Moreover, since the modification of CO_2_ levels on the ISS, there has been no change in the occurrence of SANS, making CO_2_ levels an unlikely mechanism. Furthermore, SANS-like signs have been produced in terrestrial-based head-down tilt bed rest studies with or without elevated ambient CO_2_ levels [15, 71, 72].

#### Anthropometric measurements

A study found astronauts who developed optic disc oedema and choroidal folds during spaceflight had significantly higher preflight body weight compared to those who did not (88.6 kg vs 78.6 kg, *p* = 0.02). Increased chest and waist circumferences were also observed in astronauts who developed optic disc oedema or choroidal folds [73]. This phenomenon may be attributed to the fact that larger individuals undergo a more pronounced redistribution of body fluids within their internal organs in microgravity, resulting in significant fluid shifts between the abdominal and thoracic compartments. [74] However, these findings were contradicted by a recent study that found no association between body weight or body mass index and an increase in the total retinal thickness [75].

#### Genetic predisposition

Finally, variations in 1-carbon metabolism-associated genes correlate with increased or decreased risk of SANS during LDSF [76]. Astronauts with SANS had increased blood concentrations of metabolites within this pathway before and after LDSF (albeit the concentrations remained within the normal limits for the population) [68, 77]. It is speculated that these differences account for altered nitric oxide metabolism, oxidative stress and vascular endothelial dysfunction, thereby predisposing to optic disc oedema [76, 78]. A case report of severe SANS in a female astronaut with a 1-carbon metabolic pathway genetic variant and small optic cup treated with vitamin B supplementation has been reported [79]. Several B vitamins, including vitamin B6, B12, folate and riboflavin, act as cofactors of 1-carbon metabolism. It is postulated that in SANS, particularly in those with genetic variants in the 1-carbon pathway, supplementing the 1-carbon metabolism pathway with vitamin B may be neuroprotective [2]. However, this requires further study as data supporting neuroprotection in other optic nerve diseases is conflicting.

As SANS is a constellation of clinical features that present inconsistently amongst astronauts, the processes leading to the signs may be multifactorial. Additionally, it is possible that individual risk factors exist in coupling, mechanisms to the development of SANS, such as optic cup volume and height, which have been associated with the magnitude of optic disc oedema [75]. A study of sixteen astronauts who completed two long-duration missions found that a past diagnosis of SANS was highly predictive of the same diagnosis in the subsequent long-duration mission and the severity of optic disc oedema appeared similar across both missions [19]. Identification of astronaut- specific risk factors would be of great interest to enable a future personalised medicine approach to SANS.

### Models of SANS

#### Bed rest studies

Head down tilt bed rest (HDBR) studies use gravity to simulate headward fluid shifts that occur in space. In 2018, investigators used a strict protocol of 6-degree HDBR and raised CO_2_ levels where participants were not permitted to take breaks or use a pillow to prop up their head. During this study, 45% of participants developed SANS-like optic disc oedema after 30 days [71]. Other research has shown increased nasal RNFL [80], choroidal thickness [81] and choroidal folds [72] during HDBR and found that the effect was dose dependent [72, 80]. Moreover, the same genetic variations in the 1-carbon metabolism pathway that increased risks of SANS for astronauts were associated with the risk of developing optic disc oedema in HDBR [82]. A recent HDBR study demonstrated that shorter sleep duration and lower core temperature were associated with the development of optic disc oedema [83].

The MRI scans of participants in the HDBR studies have also shown similar changes to astronauts. There was an increase in the cross-sectional area of the internal jugular vein and a decrease in the cerebral venous outflow [84, 85], upward brain displacement and increased ventricular size [86, 87]. Additionally, drainage of the lymphatic system from the head and neck was shown to be assisted by gravity in HDBR studies [88], indicating that reduced lymphatic flow in microgravity could further compound issues related to fluid shifts. Prolonged HDBR leads to worsening performance in cognitive and behavioural tasks [89, 90]. Interestingly, participants who developed SANS-like illness were shown to develop different cognitive processing strategies [91, 92] and differences in the activity of visual and vestibular cortical areas in fMRI scans [93–95].

It is worth noting that the number of participants in HDBR studies was relatively small. Nevertheless, the changes observed demonstrate the replicability of SANS findings and raise potential concerns about the possible neurological sequelae of SANS. Subtle different OCT findings have been described in HDBR and SANS [15, 96]. These findings were likely generated by gravity acting in the anterior-posterior direction during HDBR, which limits the expansion of the thoracic cage, spinal dural sac, jugular veins and the eye within the orbit [15], leading to altered fluid outflow from the head and neck. Indeed, even simply lying supine or prone can have differential effects on the eye due to the direction of gravity [97].

Despite these discrepancies, HDBR has proven invaluable for testing potential SANS countermeasures. For example, lower body negative pressure (LBNP) is used to draw blood back into the lower limb veins and has shown beneficial effects on ICP [98], intraocular pressure [99], optic nerve sheath diameter [100] and internal jugular vein volume [101]. In HDBR studies, doses of 20-25 mmHg LBNP were considered safe, whilst higher pressures led to reductions in cardiac stroke volume [102]. Furthermore, the use of LBNP for 8 hours overnight during sleep limited the expansion of the choroid on OCT [103]. In contrast, artificial gravity with short arm centrifuges for 30 minutes per day during HDBR has so far not shown improvements in intraocular pressure or OCT measures [72, 104]. Resisted inspiratory breathing devices have also been shown to reduce ICP and internal jugular vein volume during HDBR [105, 106]. Use of veno-occlusive thigh cuffs for short duration (<1 hour) was evaluated for maintaining venous blood in the lower limbs, but was less effective than LBNP at reducing internal jugular vein volume and ICP [105, 106]. Yet they are significantly more comfortable than LBNP, less complex and less expensive and therefore, may have a place in the treatment of SANS [105]. Alternatively, in HDBR studies, swimming goggles were demonstrated to increase intraocular pressure, which could help to normalise the pressure gradient across the posterior eye and alleviate SANS [107]. To enable choices to be made regarding treatment for SANS cases, it is important that future studies use clinically relevant outcomes of direct measures of changes observed in SANS, such as optic disc oedema and visual acuity, rather than surrogate markers such as intraocular pressure and jugular vein volume.

#### Terrestrial analogues

**Fig. 2:**
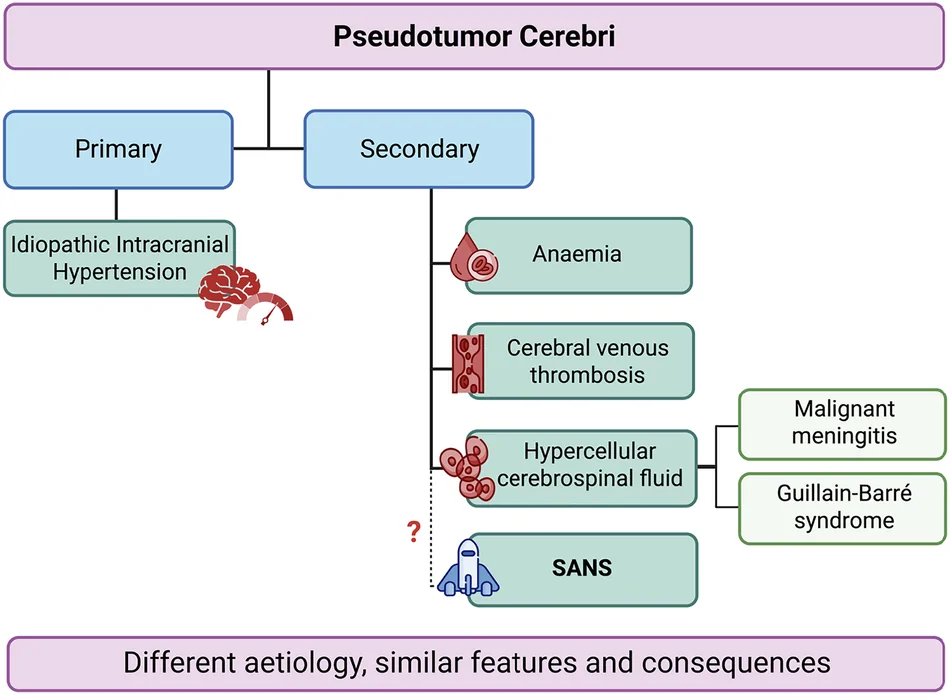
Classification of pseudotumour cerebri. Primary pseudotumor cerebri (idiopathic intracranial hypertension) is characterised by raised intracranial pressure (ICP) without an identifiable underlying cause. Secondary pseudotumor cerebri includes conditions of raised ICP due to causes such as anaemia, cerebral venous thrombosis, malignant meningitis and Guillain-Barré syndrome.

In terrestrial medicine, raised ICP in the absence of space-occupying lesions is termed pseudotumor cerebri; this can be primary or secondary. This entity presents bilateral optic nerve head oedema and increased ICP. Primary pseudotumor cerebri is also known as idiopathic intracranial hypertension. Secondary pseudotumor cerebri, also known as secondary intracranial hypertension, is defined by a condition of raised ICP due to secondary causes, which when treated, leads to resolution of papilloedema and reduction in ICP. Secondary causes of raised ICP include venous sinus thrombosis, anaemia, CSF hypercellularity (Guillain-Barré syndrome, malignant meningitis) and drug-related causes (Fig. 2). SANS likely represents a type of secondary intracranial hypertension as the condition typically resolves after cessation of microgravity exposure. However, ongoing features may persist without prompt management in both conditions. Notably, exposure to potential causative factors in pseudotumor cerebri (e.g. drug causes) does not uniformly trigger disease manifestations, echoing the variability seen in SANS, where not all microgravity-exposed individuals exhibit clinical features. Individual risk factors likely contribute to both conditions, but are not fully elucidated.

There are clear differences between IIH and SANS [49]. IIH is a disease that primarily affects young women of reproductive age and is strongly associated with obesity [108]. On the other hand, there is no clear gender based difference in SANS prevalence and susceptibility. The distribution shows similar trends for males and females [19, 75]. Interestingly, younger women with IIH are more likely to have hyperandrogenism [109], which may be a common risk factor in SANS, given pre-flight levels of the androgen dehydroepiandrosterone are associated with increased findings of SANS in astronauts [76]. In SANS, there are different patterns of chorioretinal folds on OCT imaging compared to IIH [49], which may be more consistent with a spectrum of disorders described as venous overload choroidopathy (supplemental Table 1) [58]. OCT imaging in IIH typically demonstrates anterior displacement of Bruch’s membrane, whereas studies of SANS have previously shown posterior displacement [14, 17]. More recent evidence suggests patients with SANS can exhibit either anterior or posterior displacement of the Bruch’s membrane [41]. Both SANS and IIH show changes to pituitary morphology as described above. Imaging studies have also demonstrated that cerebral transverse venous sinus stenosis (narrowing) is typically observed in IIH [110]. In contrast, a magnetic resonance venography study of 12 astronauts found significantly greater increase in venous sinus volume (superior sagittal sinus and transverse sinus) postflight in astronauts who had SANS compared to astronauts without SANS [55]. Ventricular size in IIH was initially thought to be ‘slit-like’, but this has been shown to be incorrect and more recent evidence suggests that ventricular size is normal [111]. In contrast, although there is no gross ventricular deformity in SANS, there are reports of increasing ventricular diameter [23–25, 28, 30, 34, 35, 112, 113]. Additionally, early case reports showed gross signs of SANS can present unilaterally [11, 12], whereas raised ICP is usually considered to cause bilateral papilloedema. However, more recent high-resolution data suggest bilateral optic nerve involvement in most SANS cases, contrary to earlier reports of a right-sided bias [2]. Yet asymmetric findings could be accounted for as the condition is picked up early in astronauts and is typically mild. Optic nerve head swelling asymmetry, defined as a difference of ≥2 Frisén grades between the two eyes, has also been reported in IIH; this degree of asymmetry, however, is uncommon (<4%). It appears to occur more frequently in older patients (of an age comparable to astronauts) and has been hypothesised to reflect interocular differences in optic canal anatomy. [114, 115].

There is no terrestrial disease that is a perfect model of SANS. Nevertheless, it is important to note the clinical features, typical disease course and long-term consequences of these other forms of pseudotumor in the context of gaining a comprehensive understanding of SANS. For example, profound anaemia is a known cause of secondary pseudotumour cerebri terrestrially, whilst in space flight, subtle changes in red blood cells can be noted, which may be pertinent [116, 117]. Additionally, approaches to evaluation and monitoring of biomarkers and therapies may be translatable from terrestrial pseudotumor cerebri to SANS.

### Clinical consensus guidance

#### Consensus diagnostic criteria for SANS

With the number of cases of SANS predicted to increase with the growth of LDSF by space agencies and commercial space flight, there is a growing need to publish current diagnostic criteria. NASA has put forward the following criteria to describe the categories of SANS (Table 2). However, these criteria currently do not reflect the brain manifestations and will likely evolve as our understanding of the condition advances. Clinical criteria are essential to establish a firm diagnosis and to allow for standardised clinical care as well as to enable comparison of cases in a research setting.

**Table 2:** Diagnostic criteria for spaceflight-associated neuro-ocular syndrome (SANS).

Feature of SANS	DIAGNOSTIC THRESHOLDS FOR SANS			
	*Earliest Indication*	*Clinically Concerning*	*Pathological: Acute Functional Impact*	*Pathological: Potential Long-Term Health Impact*
	Class A	Class B	Class C	Class D
**OPTIC DISC OEDEMA**	≥20 µm average increase in peripapillary total retinal thickness (TRT) and/or evidence of mild oedema, but not meeting Frisén grade 1	≥55 µm average increase in peripapillary TRT and/or Frisén grade ≥1	Visual field loss (e.g. enlarged blind spot) *Associated anatomical signs to be determined*	Permanent visual field loss and/or reduced retinal nerve fibre layer (RNFL) thickness
**CHORIORETINAL FOLDS**	Any evidence of folds (choroidal, retinal, or peripapillary wrinkles)	Sharp folds in the vicinity of the macula	Distorted central vision *Associated anatomical signs to be determined*	Permanently distorted central vision, atrophy of retinal pigment epithelium (RPE) or photoreceptors and/or choroidal neovascularisation
**GLOBE FLATTENING**	Any evidence of posterior globe flattening (centred at optic nerve insertion) or decreased axial length (centred at fovea)	Moderate change	Currently unknown	Currently unknown
**REFRACTIVE ERROR SHIFT**	> +0.50 Dioptres	Shift in refractive error approaching the power limit available in-flight Space Anticipation Glasses	Shift in refractive error beyond the power limit of available in-flight Space Anticipation Glasses	Currently unknown

Classification of SANS based on severity and features. Only one of the criteria is required to meet each stated classification. Optical coherence tomography values obtained from Spectralis^TM^ Heidelberg [19].

*TRT* total retinal thickness, *RNFL* retinal nerve fibre layer, *TPE* retinal pigment epithelium.

An average increase of 20 µm in peripapillary total retinal thickness (TRT) on OCT imaging (Spectralis^TM^ Heidelberg Engineering) is used as the earliest indication threshold to detect optic disc oedema, with a low chance of sampling error or physiological variation [15, 118]. Using this threshold, 66% of astronauts after LDSF may be diagnosed with optic disc oedema due to SANS (Table 2).

#### Monitoring of SANS

A consensus for how to manage and monitor SANS is not fully established; it is an ever-evolving subject. Evaluating for SANS onboard the ISS is time-consuming in a time-critical environment and may impact other mission tasks. To monitor SANS in greater detail, astronauts are now trained to perform orbital ultrasound, fundus imaging and OCT imaging in orbit. However, the added value of each testing modality needs to be considered, weighed against its impact on crew time. For example, the ISS OCT provides an infrared en face and MultiColor Imaging capabilities that are used in lieu of traditional colour fundus photography under nominal conditions, thus avoiding the time required to perform a separate fundoscopy session. The current high-density scan protocols for OCT imaging can be time-consuming. If screening for SANS is the main aim, then extensive protocols could be streamlined for the purpose of disease monitoring alone. Although this would be at a cost of reduced data, which could elucidate how the ocular features develop in SANS. Visual field assessment capability was deployed to ISS in late 2025 and is now available for testing clinically concerning SANS cases.

**Fig. 3:**
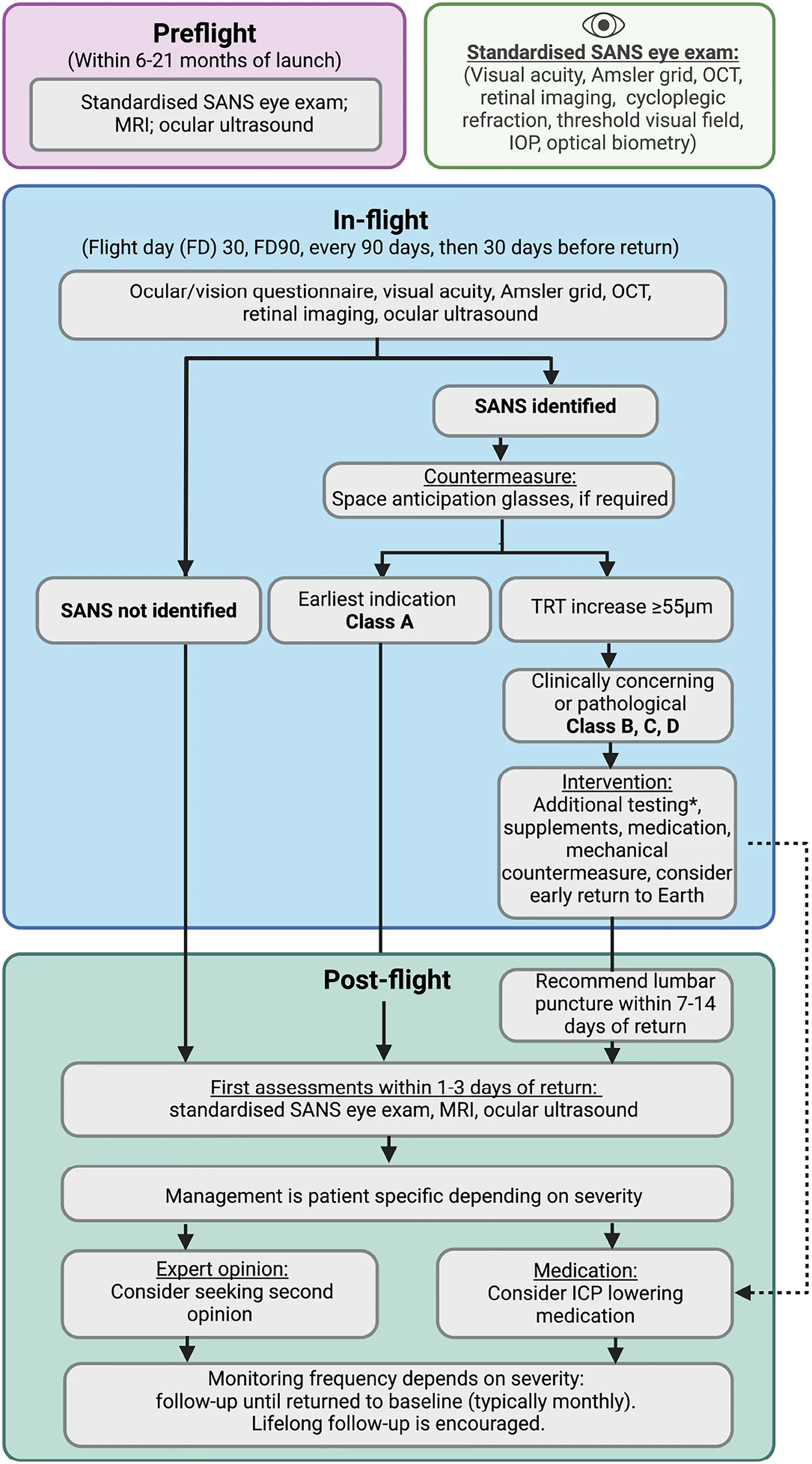
Consensus management overview for spaceflight neuro-ocular syndrome (SANS). Adapted from the primary testing and decision tree overview for spaceflight-associated neuro-ocular syndrome (SANS) as applied to NASA astronauts assigned to long-duration missions onboard the International Space Station (ISS). MRI magnetic resonance imaging, FD flight day, OCT optical coherence tomography, TRT total retinal thickness, IOP intraocular pressure, ICP intracranial pressure. *More frequent assessments may be required, depending on severity or rate of progression.

Current protocols at NASA stipulate routine SANS/ocular evaluations before and after a mission to the ISS; on mission days 30 and 90; and 30 days before landing for 6-month missions. MRI is also routinely performed before and after ISS missions (Fig. 3). Once a diagnosis of SANS is made, the severity of disease can be classified (Table 2) with the appropriate measurements made (supplemental Table 2). The frequency of ongoing OCT monitoring should be based on the risk to the astronaut with the condition grade and rate of the increase in optic nerve oedema balanced against the time taken for monitoring against other mission deliverables. Post-mission ongoing monitoring should occur until the features are resolved. Crew members meeting criteria for class B and beyond, or those with an increase in TRT of ≥55 µm are currently recommended to have a post-flight lumbar puncture (Fig. 3).

### Treatment of SANS

#### Currently utilised countermeasures

To date, there are no established treatments for SANS beyond utilising space anticipation glasses for suspected shifts in refractive error. For individuals affected by SANS, respite from the disease comes only after terrestrial return on completion of the mission. Until effective countermeasures are established, individuals with pre-existing neuro-ophthalmological conditions such as idiopathic intracranial hypertension or glaucoma should be cautioned from participating in commercial or other spaceflight activities. Furthermore, individuals with previous SANS may need enhanced monitoring during future missions, depending on the grade of the SANS and recovery of the features [19].

Russian cosmonauts undergo lower body negative pressure (LBNP) sessions to prevent cardiovascular adaptations during LDSF during the final 21 days of missions. This was shown to reduce ICP in HDBR studies as discussed above and may prove effective in SANS. Short-duration LBNP on the ISS has been shown to reduce internal jugular vein area, middle cerebral vein velocity and intra-ocular pressure to terrestrial levels [60, 119], but did not improve measures on OCT imaging, suggesting longer sessions may be required [17, 119]. Current machines are impractical and if long durations of LBNP (8 h) are required, this will limit astronauts in undertaking important mission tasks. A recent HDBR study found that wearing veno-constrictive thigh cuffs (VTC) for 6 h after an hour of aerobic exercise to augment blood flow to the legs leads to a transient reduction of the internal jugular vein cross-sectional area and is well-tolerated, suggesting its potential use as a countermeasure [120].

When SANS was first recognised, exercise, specifically the Valsava manouvre and breath-holding during resistive activity, was evaluated to ensure the astronauts were breathing/exhaling and not breath-holding during resistive exercise in case this was contributing to raised ICP. Although there was no reliable method to measure compliance or verify accuracy, the Astronaut Strength and Conditioning Rehabilitation Specialists (ASCRs) continued to teach and monitor the astronauts’ exercise regimens on the ground to support proper training.

Another possible countermeasure is a device (like swimming goggles) that mildly raises intraocular pressure to similar levels to ICP, hence minimising the translaminar pressure gradient and permitting axoplastic flow. This would be a device similar to the Ocular Pressure Adjusting Pump (OPAP), where positive pressure would be applied to the periocular space, rather than negative pressure. This type of countermeasure might mitigate the ocular signs of SANS, but likely would not affect the brain changes associated with long-duration spaceflight.

#### Emerging therapeutic strategies

Medications to reduce ICP and CSF volume may be a realistic management option for SANS. Drugs such as acetazolamide and topiramate are widely used to reduce CSF secretion (and consequently ICP) and optic nerve oedema in IIH [121, 122]. Although one of the original SANS cohort was briefly treated post-flight with acetazolamide [5], the side effect profile includes cognitive dysfunction [3], acidosis and increased risk of renal stones. These would not be acceptable risks during LDSF missions. Topiramate is not an option in astronauts as it causes significant cognitive impairment at therapeutic doses [123].

In rat models, glucagon-like peptide 1 (GLP-1) receptor agonists were found to alter CSF secretion within the brain and reduce ICP via reduction of Na+ and K+ dependent ATPase activity, a key regulator of CSF secretion [124]. A recent randomised clinical trial demonstrated that exenatide, a GLP-1 receptor agonist, significantly reduced ICP in patients with active IIH from as early as 2.5 h, with effects seen at 24 h and 3 months with continued dosing. The drug is well-tolerated, typically causes nausea in the first week, with no serious adverse events noted [125]. Additionally, studies have demonstrated that GLP-1 receptor agonists have well-established safety profile in individuals with obesity [126]. If GLP-1 receptor agonists were to be repurposed for SANS, this could be used prophylactically or following diagnosis. Weight should be monitored and a flexible-dosing approach could be employed to mitigate unintended weight loss [127]. Further prospective evaluation is warranted.

### Limitations

We recognise that the evidence may be limited due to the small sample sizes in the astronaut studies and some of the studies include astronauts with repeat spaceflight experience. When the evidence is sparse, some recommendations are based on expert opinion, which is inherently subjective. This consensus guidance will need updating periodically to reflect emerging evidence.

## Conclusion

In summary, SANS constitutes a constellation of features that are typically mild during 6-month missions. It is diagnosed in males and females, in novice and veteran astronauts and in either or both eyes. Minimal changes attributed to SANS are observed almost ubiquitously during spaceflight. Severe manifestations of SANS have been rare and no permanent loss of vision or cognitive function has yet been detected in any crew members. However, it is predicted that SANS will increase in prevalence and severity as extended duration spaceflight (i.e., beyond LDSF) is pursued. This consensus report details SANS diagnostic criteria, stages of disease severity and guidance on potential treatment avenues. The risk factors for developing severe SANS are not known but represent an important area of future study. There is a need to establish objective, internationally recognised criteria for the measurement of disease features and to decide at what level astronauts should be treated. Countermeasures and treatment are currently an unmet clinical need in SANS and it is critical to determine the underlying mechanisms in SANS pathogenesis, including the possible role of elevated intracranial pressure. Advances in CSF disorders in terrestrial medicine from the use of OCT imaging to track disease and the use of GLP-1 receptor agonists to reduce ICP might be of potential use in SANS.

## Summary

### What is known about this topic

- Spaceflight‑Associated Neuro‑Ocular Syndrome (SANS) is a recognised risk of long‑duration spaceflight, characterised by optic disc oedema, globe flattening, choroidal folds, and refractive changes.
- Pathophysiology remains uncertain, but proposed mechanisms include cephalad fluid shifts, altered CSF dynamics, and venous congestion in microgravity.
- Idiopathic intracranial hypertension (IIH) shares some overlapping clinical and imaging features with SANS, including raised ICP‑like signs without a mass lesion.
- There is a lack of effective countermeasures to date. Lower body negative pressures and fluid redistribution techniques are under evaluation.

### What this study adds

- Expert consensus integrating NASA SANS specialists and experts of terrestrial CSF disorders provides the first harmonised framework for diagnosis and management for SANS.
- Diagnostic threshold for SANS.
- Operational recommendations for in‑flight monitoring and post‑flight follow‑up.

## Supplementary information

Supplemental Table 1, Supplemental Table 2
