# Supplementary material for: Spaceflight-associated neuro-ocular syndrome (SANS): expert consensus on diagnosis and management
Source: Eye (Lond). 2026 Jun 30;40(12):1794–805. doi: 10.1038/s41433-026-04651-6 (PMC13462731; doi:10.1038/s41433-026-04651-6)
Supplement: Supplementary file 1 [file 41433_2026_4651_MOESM1_ESM.docx]

**Supplemental table**

| **Condition** | **Symptoms** | **Ophthalmic examination** | **Magnetic resonance imaging (MRI) features** | **Intracranial pressure (cmH_2_O)** |
| --- | --- | --- | --- | --- |
| **Spaceflight associated neuro-ocular syndrome**  **Demographics:**  M=F  Any age | Hyperopic acuity shift  Occasional mild headaches (usually associated with sinus congestion or elevated CO_2_ levels) | Optic disc oedema (mild)    Posterior globe flattening  Posterior displacement of Bruch’s membrane  Choroidal folds more prevalent; typically concentric with linear component towards macula | Mild pituitary concavity  Enlarged optic nerve sheath  Ventricular expansion  Upward brain shift  Distended venous sinuses | 18-28.5 |
| **Head Down Tilt Bed Rest**  **Demographics:**  N/A | None | Optic disc oedema    Chorioretinal folds    No choroidal thickening | Ventricular expansion    Upward brain shift | Acutely increased proportional to degree of tilt |
| **Idiopathic Intracranial Hypertension**  **Demographics:**  F > M  Median age at diagnosis: 28 years | Prominent chronic headaches  Pulsatile tinnitus  Transient visual obscuration | Optic disc oedema  Posterior globe flattening  Anterior displacement of Bruch’s membrane    Retinal folds more prevalent; typically concentric | Severe pituitary concavity and empty sella  Enlarged optic nerve sheath  Normal or small ventricles  Transverse venous sinus stenosis | >25 for diagnosis, often >30 |
| **Venous overload choroidopathy**  **Demographics:**  M > F  Age: 30-50 years | May have hyperopic acuity shift | Optic disc oedema    Posterior displacement of Bruch’s membrane    Choroidal folds more prevalent    Pigment epithelial detachment | May be normal depending on the etiology | >25 |
| **Hydrocephalus**  **Demographics:**  M = F  Age >60years | Headache  Gait disturbance  Cognitive decline  Urinary incontinence | May be normal | Ventricular expansion | May be normal or raised depending on aetiology |

Supplemental Table 1: Findings of spaceflight associated neuro-ocular syndrome (SANS) as compared to other terrestrial analogues and pathologies with similar manifestations. M = male; F = female; N/A = not available.

| **Features** | **Measurement tool** |
| --- | --- |
| Optic disc oedema: Peripapillary total retinal thickness (TRT) | Optical coherence tomography (OCT) (Spectralis^TM^ Heidelberg engineering) |
| Optic disc oedema: Frisén grading | Fundoscopy and/or OCT MultiColor Imaging |
| Chorioretinal folds | OCT imaging qualitative examination, fundoscopy and/or OCT MultiColor Imaging |
| Posterior globe flattening | Magnetic resonance imaging (performed pre- and post-flight only) and ultrasound |
| Axial length | Optical biometry (performed pre- and post-flight only) |
| Refraction shift: pre- to post-flight | Cycloplegic refraction (performed pre- and post-flight only) |
| Refraction shift: in flight | Estimated by change in visual acuity, vision questionnaire responses, and use of individualised Space Anticipation Glasses |
| Visual field | 24-2 SITA Standard (performed pre- and post-flight only) and Amsler gird |

Supplemental Table 2: Spaceflight associated neuro-ocular syndrome (SANS) clinical features as measured by the following tools. Optical coherence tomography values obtained from Spectralis^TM^ Heidelberg. TRT = total retinal thickness. Refraction is established pre mission and used for the visual field.
